# The Diagnosis of Common Variable Immunodeficiency After Multisystem Dysfunction

**DOI:** 10.7759/cureus.21624

**Published:** 2022-01-26

**Authors:** Taha F Rasul, Daniel R Bergholz, Arfa Faiz

**Affiliations:** 1 Department of Infectious Diseases, University of Miami Miller School of Medicine, Miami, USA; 2 Department of Allergy and Immunology, University of Miami Miller School of Medicine, Miami, USA; 3 Allergy and Immunology, Sutter Medical Center, Sacramento, USA

**Keywords:** immune dysfunction, multi-disciplinary care, recurrent infection, non hodgkin's lymphoma, delayed diagnosis, common variable immunodeficiency deficiency

## Abstract

Common variable immunodeficiency (CVID) is a primary immunodeficiency caused by the lack of B cell differentiation into plasma cells, thereby leading to decreased serum immunoglobulins. Patients with this condition are predisposed to recurrent infections and are more likely to develop certain cancers and autoimmune diseases. We report the case of a 53-year-old female suffering from recurrent pulmonary infections and a history of non-Hodgkin lymphoma (NHL) who had a poor response to the measles, mumps, and rubella (MMR) and varicella vaccines as a child, and was infected with coronavirus disease 2019 (COVID-19) twice in 2020. Testing of her antibody titers in order to determine suitability for *Streptococcus pneumoniae (S. pneumoniae) *vaccination found an overall decrease in major immunoglobulin classes (IgG, IgM, and IgA) and B cells with normal morphology. The diagnosis of CVID was made, and prompt treatment with intravenous immunoglobulins (IVIG) brought her IgG levels up from 282 to 680 mg/dL within three months. This case highlights the importance for providers to keep immunological dysfunction on their differentials for patients with atypical presentations involving multiple organ systems.

## Introduction

Common variable immunodeficiency (CVID) is a primary B cell immunodeficiency characterized by a lack of differentiation of B cells into plasma cells; it is the most common immunodeficiency among adults. It can affect multiple organ systems and manifests a spectrum of presentations. This leads to a paucity of immunoglobulin production, causing decreased serum levels of at least two immunoglobulin classes including IgG and IgM or IgA. It is important to note that hematological evaluation often shows normal B cell morphology and number, and hence CVID is usually a diagnosis of exclusion. Early detection of CVID is crucial because it can predispose a patient to many diseases later on in life.

Due to the remainder of the immune system staying intact, and given the diagnosis of exclusion aspect, CVID is typically diagnosed after puberty and often presents with recurrent pyogenic respiratory infections [1,2]. A typical patient presentation involves diffuse lymphadenopathy, enlarged tonsils, and/or splenomegaly. Another important consideration is that CVID predisposes patients to the development of non-Hodgkin lymphoma (NHL), gastric cancer, and autoimmune disorders (such as various cytopenias and rheumatoid arthritis) [3]. The immune dysregulation found in CVID can present as a multitude of disorders, ranging from chronic lung disease to granulomatous infiltration of various organs [4]. Pulmonary symptoms are by far the most common ones, and some studies have shown that roughly one-third of patients have chronic lung disease by the time they are diagnosed [5]. These can take the form of either obstructive or restrictive lung disease, and injury typically occurs due to repeated, chronic infections [6,7]. Moreover, the knowledge of predisposition towards malignancy development is especially crucial as it can guide preventative treatment. Therefore, it is vital for clinicians to keep CVID on their differentials for patients with lung disease, gastrointestinal disease [presumably due to a blunted immune response towards pathogens like *Helicobacter pylori* *(H. Pylori)*], and autoimmune syndromes [8-10].

In this report, we present the case of a 53-year-old female evaluated for multiple drug allergies. She had a history of gastroesophageal reflux disease (GERD), NHL (diffuse large B cell lymphoma), and type II diabetes mellitus. In addition to a past medical history of multiple episodes of pneumonia, she had tested positive for coronavirus disease 2019 (COVID-19) twice in the same year and also had a childhood history of poor immune response and protection from measles, mumps, and rubella (MMR) vaccine.

## Case presentation

The patient was a 53-year-old female who had an extensive medical and surgical history. Most notably, she was being evaluated for multiple self-reported allergic reactions to a wide array of drugs, specifically antibiotics (Table 1). It should be noted that these reactions were not directly demonstrated by a comprehensive allergic or immunologic workup, nor were they confirmed with skin tests. They were based on the patient's self-reported history as well as evaluations from non-immunologist providers.

**Table 1:** Previous allergies and reactions to medication

	Medication	History of allergy/reaction
Antibiotics	Penicillin	She reported strep throat and scarlet fever as a child (seven years old). After hospitalization and penicillin administration, she felt like she could not breathe. Her mother told her to unequivocally avoid penicillin afterward
	Bactrim double-strength (TMP-SMX)	Rash, urticaria, flushing, and diffuse redness over the body. There was also lip swelling
	Ciprofloxacin	In 2015, she had one pill and four hours later had swollen lips but no other major swelling or breathing difficulty. She was later told that it was a mild reaction and has been able to take levofloxacin without problems
	Cephalexin	No significant allergy to cephalexin noted
	Doxycycline	A few hours after taking the medication, she noted lip swelling and itching. One day after, she noticed her entire face was swollen. After taking diphenhydramine, her symptoms improved significantly
	Clindamycin	Combined administration during hospitalization in 2018/2019. The patient felt like her skin was peeling off and burning
Pain medication	Dilaudid	
	Morphine	Administered as an injection when she was younger; she immediately started vomiting repeatedly
Diuretics	Hydrochlorothiazide	High sensitivity to the drug, became dehydrated and could not adequately rehydrate

**Figure 1:**
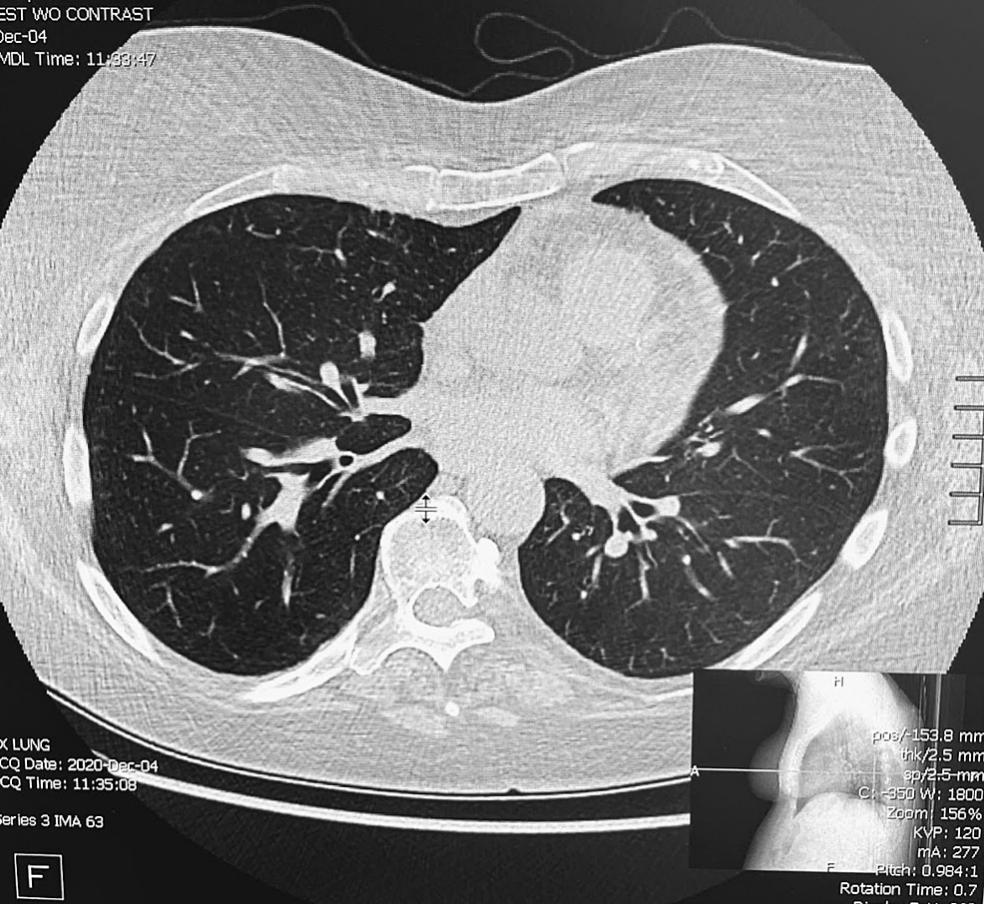
CT image of pulmonary post-infectious changes. The presence of lymphadenopathy and calcified nodules is also noted CT: computed tomography

The patient’s past medical history was extensive and included an array of diseases and interventions. She suffered from gastroesophageal reflux disease and type 2 diabetes mellitus. Most significant, however, was her history of treated NHL (diffuse large B cell lymphoma) in extranodal and solid organ sites from late 2014 to mid-2015. Additionally, she had been infected with COVID-19 in October 2020, although this had been the second infection. She had reported a previous infection with COVID-19 in late February 2020. It is not known what specific COVID-19 variant was prevalent during October 2020, particularly in the Sacramento area. Her first case of severe pneumonia had been in 2009, and she had experienced recurrent episodes shortly after chemotherapy (November 2014-May 2015), as well as in late 2020. According to patient records, her absolute leukocyte and neutrophil counts had been within normal limits prior to the later infections. An example of the extent of parenchymal inflammation and mediastinal lymphadenopathy noted in December 2020 is shown in Figure 1.

Longitudinal chest CT scans and radiographs from 2018 to 2020 showed extensive and chronic inflammatory findings over a number of years (Table 2). There was also a history of a sinus infection in 2019. Perhaps the most crucial part of the medical history was the fact that our patient had suffered from measles and mumps as a child despite receiving adequate vaccination.

**Table 2:** Imaging history from June 2018 to the present CT: computed tomography; COVID-19: coronavirus disease 2019

Date of imaging	Imaging type	Pertinent findings
12/04/2020	CT chest	Resolved scattered bilateral ground-glass opacities. No further pneumonia. Calcified lung nodules present and reactive changes such as mediastinal lymphadenopathy were noted
10/17/2020	CT angiogram chest	No apparent filling defects within central pulmonary arteries. Peripheral pulmonary arteries were suboptimal; there may have been the presence of small peripheral pulmonary thromboemboli. Bilateral ground-glass opacities suspicious for multifocal pneumonia were also consistent with the patient’s positive COVID-19 status
07/07/2020	CT chest	Increased number of nodules in the left lower lobe. Larger heterogeneous opacities in the right upper lobe. Overall findings indicative of atypical infection
03/17/2020		Multiple new bilateral small lung nodules up to 6 mm in diameter. There was also the presence of new, larger nodules >1 cm in the right upper and lower lobes. The left axillary lymph node was unchanged. There was an incidental finding of hepatic steatosis, a small accessory spleen, and a small right adrenal benign adenoma
04/03/2019	X-ray	Patchy atelectasis or focal consolidation in the right middle lung
11/12/2018	CT chest	Resolution of most of the previously seen left lower lobe nodules. Multiple new bilateral pulmonary nodules up to 1.3 cm in diameter. Ground-glass opacification suggests inflammatory or infectious etiology. Unchanged enlarged left axillary lymph node. Splenomegaly noted
06/05/2018		Scattered nodules and subpleural ground-glass opacities. Subtle left lower lobe consolidation, possibly indicative of atypical infection. Unchanged enlarged left axillary lymph node

The patient's physical exam was most notable for post-nasal drip as well as joint pain and paresthesias in both hands. There was a history of lung and colon cancer in her maternal grandfather and maternal aunt, respectively. Her past surgical history was mostly non-contributory with the exception of a deep cervical lymph node removal for biopsy in 2015, to monitor cancer remission. The findings mentioned above raised suspicions for some form of immune-system dysregulation with the most apparent problem being recurrent pulmonary infections. The fact that she mentioned having been infected twice with COVID-19 was especially alarming. A plan was made to determine if she was a suitable candidate for the *Streptococcus pneumoniae (S. pneumoniae) *PN-23 vaccine. To that end, her serum immunoglobulin titers were measured (Table 3).

**Table 3:** Serum immunoglobulin titers

Immunoglobulin isotype	Normal range (mg/dL)	Observed range (mg/dL)	Overall level
IgA	87-474	<15	Low
IgG	681-1,648	250	Low
IGG 1	382-929	147	Low
IGG 2	241-700	49	Low
IGG 3	22-178	83	Normal
IGG 4	4-86	<0.5	Low
IGG, Serum	600-1,640	259	Low
IgM	48-312	22	Low

Furthermore, during lymphocyte testing, it was found that the CD19+ cells and the absolute CD3 count were in the lower-normal range: below 100 cells/µL and 800 cells/µL, respectively. Diphtheria antitoxoid titers were <0.10, which was significantly lower than expected in a vaccinated individual.

## Discussion

After reviewing our patient's medical and imaging history, there was a high suspicion concerning CVID or some other immunopathology. However, we found the ostensible link between the patient’s past and her current health condition only by definitively measuring serum immunoglobulin titers. As seen in Table 1, there was a generalized decrease in serum titers of IgG, IgA, and IgM. The dramatically reduced levels of IgA can explain a lack of mucosal resistance to pathogens and the patient’s increased susceptibility towards sinopulmonary infection [11]. The low IgG titers also meant that not only would the patient’s immune system struggle against pathogens introduced into the bloodstream acutely, but it would also have a blunted response towards vaccination, thereby leading to chronic susceptibility to preventable diseases [12].

The overall decrease in immunoglobulin titers was also coupled with a low-normal level of CD19+ cells, where CD19 is the serum marker for B cell lineage. Coupled with the history of malignancy, immunodeficiency, and recurrent infections, CVID was deemed the most likely diagnosis for this patient.

One other pertinent immunodeficiency disorder with an overall decreased number of serum immunoglobulins is Bruton agammaglobulinemia. However, this disease manifests as a complete deficiency of mature B cells. The loss of one complete arm of the adaptive immune response causes recurrent pyogenic infections early in life (after six months of birth), especially by enteroviruses and encapsulated bacteria. Even though it is an X-linked disorder, there have also been cases noted of female agammaglobulinemia due to highly skewed X-chromosome inactivation [13]. We could rule out Bruton agammaglobulinemia in our patient because she did not have any history of severe infections very early in life, and her B cell titers were on the low end of normal.

Although our patient’s initial complaint pertained to multiple drug allergies, careful testing revealed that she most likely had undiagnosed CVID. The first concerning sign was the medical history of the patient. Recurrent sinopulmonary infections with a history of NHL should have raised concerns about perhaps some form of immunologic or hematologic dyscrasia [14]. Moreover, a childhood history of measles despite receiving the MMR vaccine warranted a definitive investigation of the overall condition of the patient’s immune system. It is now understood that contracting COVID-19 twice in the span of a few months is much more indicative of immune system dysregulation rather than mutagenicity of the virus itself [15]. In such patients, it is vital to further explore the potential factors leading to this increased susceptibility to disease.

We would like to emphasize the importance of keeping CVID and other immunodeficiencies on the differential list for a few main reasons. Firstly, numerous studies have found that CVID tends to predispose patients towards the development of NHL [16]. Therefore, early detection and treatment of CVID can enable better monitoring and response to malignancy. Additionally, our patient’s quality of life had been significantly and adversely impacted by her untreated and undiagnosed condition. Recurrent infections and lung damage had continued to occur even after her treatment for cancer. Approaching the patient’s illnesses from a holistic perspective and focusing on the patient rather than the disease can also help clinicians in recognizing the distinct pattern in the history of such patients. Although roughly one-third of patients are diagnosed with CVID before the age of 10 years, delayed identification of this disease is common [17]. It is only after multiple specialist visits that the diagnosis is usually made. For a disease with such extensive organ involvement, CVID is often overlooked, which can impede timely treatment and evaluation.

Our patient initially complained of drug sensitivity and allergies. With the diagnosis of CVID, her treatment course was guided towards addressing the immunodeficiency rather than answering the questions related to the drug allergy. Even so, it may not seem immunologically likely that the drugs mentioned with different mechanisms of action would produce the same symptoms, such as lip swelling and facial redness [18]. It is important to note that formal allergy testing was not conducted to confirm any of the mentioned drug allergies. As such, hypersensitivity against drugs was not appropriately assessed.

She was treated with intravenous immunoglobulins (IVIG) infusions, which raised her IgG levels up to 680 mg/dL from 282 mg/dL within three months. Treatment was provided at a dose of 500 mg/kg infused every four weeks. The patient was counseled on her condition and she expressed relief finally knowing that she may have a diagnosis that explains her multitude of symptoms. At the follow-up six months later, her immunoglobulin titers were still within normal limits and she reported subjective improvement in terms of decreased sinusitis over that time frame.

## Conclusions

Our patient's extensive history of diminished vaccine response, recurrent sinopulmonary infections, and neoplastic disorders were likely caused by her undiagnosed CVID. Due to the multitude of organ system involvement and late presentation, clinicians may treat such illnesses as separate entities rather than as consequences of a singular condition. It is important to keep CVID and other immunodeficiencies on the differential list even if there are multiple unrelated symptoms and a history of recurrent infections. Timely diagnosis and management can ideally provide patients closure and a better understanding of their health.
